# Effects of the Spatial Structure Conditions of Urban Underpass Tunnels’ Longitudinal Section on Drivers’ Physiological and Behavioral Comfort

**DOI:** 10.3390/ijerph182010992

**Published:** 2021-10-19

**Authors:** Zhongxiang Feng, Miaomiao Yang, Yingjie Du, Jin Xu, Congjun Huang, Xu Jiang

**Affiliations:** 1 School of Transportation, Southeast University, Nanjing 210096, China; fzx@hfut.edu.cn; 2 School of Automobile and Traffic Engineering, Hefei University of Technology, Hefei 230009, China; rajondu@163.com; 3 College of Traffic and Transportation, Chongqing Jiaotong University, Chongqing 400074, China; yhnl_996699@163.com; 4 Hefei Urban Planning and Design Institute, Hefei 230009, China; hcj13965051002@outlook.com (C.H.); jiangxu_524@live.com (X.J.)

**Keywords:** traffic safety, urban underpass tunnel, driving simulator experiment, physiological characteristics, tunnel height, slope, slope length

## Abstract

To investigate the physiological and behavioral comfort of drivers traversing urban underpass tunnels with various spatial structure conditions, a driving simulator experiment was conducted using 3DMAX and SCANeRTM studio software. Three parameters, including the slope, slope length, and height of a tunnel, were selected as research objects to explore the optimal combination of structural parameters in urban underpass tunnels. The heart rate (HR), interbeat (RR) interval, speed, and lane centerline offset value were collected for 30 drivers. Then, a measurement model of the relationship among HR, RR interval, speed, lane centerline offset value, and structural parameters was established by using partial correlation analyses and the stepwise regression method. On this basis, a structural constraint model based on the drivers’ physiological and behavioral comfort thresholds was also constructed. The results show that the driver’s HR, RR interval, speed, and lane centerline offsets are significantly related to the tunnel height, slope, and slope length. More importantly, this paper not only analyzed the effects of various structural parameters on drivers’ physiology and behavior but also proposed an optimized combination of structural parameters based on drivers’ physiological and behavioral comfort. It can reasonably improve tunnel design in China, ensure tunnel traffic safety, and seek the maximum comfort of the driver in the driving process.

## 1. Introduction

The conflict between rapid urban expansion and limited land resources is intensifying. The development of underground space has become necessary to solve this contradiction. Therefore, many urban underpass tunnels have emerged. Although urban underpass tunnels play a positive role in reducing urban road land, decreasing driving mileage, and dredging urban traffic, there are also many potential safety hazards. Due to the difficulties in rescue and on-site evacuation for tunnel accidents, almost every accident causes large-area and long-term congestion, resulting in immeasurable economic losses and very serious social impact. Tunnel safety is an important subject that has attracted the attention of many traffic scholars. Studies have shown that the accident rate in tunnels is lower than that on ordinary roads, but the mortality rate is higher [1,2,3,4]; thus, the consequences of these accidents are very serious [5,6]. Therefore, the traffic safety of urban underpass tunnels needs to be widely considered.

In fact, urban underpass tunnels are a special form of highway tunnel that conform to the tunnel form in terms of structure and function and have many similarities with ordinary highway tunnels. Therefore, urban underpass tunnel designers often refer to relevant specifications and standards, such as “code for design of highway tunnel”, “code for design of urban road”, or “code for design of urban underground road engineering”, when designing parameters. Although such documents give comprehensive consideration to tunnel civil engineering, they do not give sufficient consideration to road traffic safety and lack a certain rationality. There are major differences between ordinary road tunnels, urban roads, and urban underpass tunnels. The structure of urban underpass tunnels is clearly distinguishable from that of ordinary road tunnels, which are typically composed of a tunnel entrance, a middle section, and a tunnel exit. In urban underpass tunnels, the entry section is typically oriented downhill, the exit section tends to be oriented uphill, and the middle section is gently concave. Relative to ordinary urban roads, the driving environment in urban underpass tunnels tends to include relatively narrow lanes, a traffic light at the tunnel entrance, variations in the height of tunnel walls, a narrower driving space, and a limited line of sight. These environmental factors can aggravate the effects of the tunnel structural parameters on tunnel traffic safety. Therefore, if the design parameters of urban underpass tunnels are based on specifications for highway tunnels and urban roads, they will not meet the safety requirements for urban underpass tunnels and may lead to serious security risks. 

In addition, for the horizontal and vertical alignment of the tunnel portal and the connection between the portal and the route outside the tunnel, there is great fuzziness in the regulations, which makes many design units prone to traffic safety accidents at the portal after the completion of the tunnel, although the design standards adopted in the tunnel survey and design are appropriate [7]. Urban underpass tunnels are typically composed of a tunnel entrance, a middle section, and a tunnel exit. In urban underpass tunnels, the entry section is typically oriented downhill, the exit section tends to be oriented uphill, and the middle section is gently concave. However, in China, the slope, slope length, and height of urban underpass tunnels are based on standards designed for highway tunnels and urban roads. Generally, a tall tunnel should have a long slope length to avoid excessive sloping; this may be considered a waste of urban land resources. However, shorter tunnels restrict vehicle traffic and affect traffic safety. Therefore, it is difficult to design reasonable structural parameters for urban underpass tunnels. According to Huang [8], data on 262 typical Chinese urban tunnel traffic accidents indicate that the accident rate in longitudinal sections of urban underpass tunnels is as high as 50%. Wei et al. [9] pointed out that the accident rate of uphill sections increases with slope length.

Research shows that approximately 90% of traffic accidents are caused by human factors [10,11]. Moreover, Ren [12] proposed in the early 1990s that the current theoretical specifications related to road design based on “vehicle driving theory” ensure only the minimum safety of vehicles in kinematics but fail to fully consider the psychological and physiological comfort of road users. Huvarinen et al. [13] pointed out that only complying with automobile road design and construction standards cannot ensure traffic safety and that considering the driver’s physiology and behavior can minimize the accident risk. Du [14] pointed out that behavior is the physical and mental changes of people produced by facing environmental stimuli, which play a guiding role in the design of architectural space and have an important influence on the entire architectural design process. Therefore, from the perspective of drivers’ physiological comfort, it is necessary to explore the “rationality” of the structural parameter design of urban underpass tunnels to improve the traffic safety of urban underpass tunnels.

Generally, the road alignment design of urban underpasses in China mainly considers vehicle performance and trafficability, ignoring the psychological and physiological characteristics of drivers. Therefore, combined with ergonomics and aiming at considering driver comfort, this paper analyzed the optimal parameters of urban underpass tunnels to contribute to the reasonable improvement of urban underpass tunnel design in China and further promote tunnel safety.

## 2. Literature Review

### 2.1. Drivers’ Physiological and Behavioral Characteristics and Tunnel Safety

Regarding tunnel safety, the relationship between drivers’ physiological and behavioral characteristics and tunnel safety has aroused much attention from scholars. The drivers’ electrocardiogram (ECG) and speed variation characteristics in various sections of the tunnel (entrance, exit and internal section) and the visual variation characteristics of drivers at the entrance and exit of the tunnel [15,16,17,18,19,20] and tunnel environment [21,22,23] have been intensively discussed in the field of tunnel safety. For instance, the “black hole” and “white hole” effects caused by the rapid change in illumination at the tunnel entrance and exit have an impact on drivers’ visual adaptability [24,25,26]. Verwey [27] took heart rate (HR), blink times, skin electrical response, respiratory rate, driving speed, and steering wheel reversal as indicators to explore changes in the physiological characteristics and handling behaviors of drivers at a tunnel entrance. Qi et al. [28] used heart rate growth (HRG) as the index to classify the driving risk level. Miller et al. [29] used driver HR and the standard deviation of the cardiac interval to explore the relationship between driver stress and driving performance. The spatial changes in a driver’s HR growth rate, root mean square of successive RR interval differences (RMSSD), pupil diameter growth rate, and vehicle lateral deviation within 300 m before and after the tunnel entrance and exit were analyzed to determine the variation rules in various tunnels [30]. Qin et al. [31] discussed the impacts of color decorations on interior tunnel walls on improving the constant speed driving time and the lane offset. Zhao et al. [32] mainly analyzed the variation characteristics of driver HR and speed at the entrance of a long tunnel and quantified the relationship between driver HR and speed. Liu et al. [33] pointed out that the light environments of a tunnel have a significant impact on the change in driver HR. 

### 2.2. Longitudinal Slope and Drivers’ Physiological and Behavioral Characteristics

As an important structural parameter, the influence of slope on critical ventilation velocity in tunnels and tunnel evacuation in the case of fire [34,35,36,37] and the relationship between slope and driving load are also hotspots of tunnel safety research. Wang et al. [38] pointed out that the average fixation time and visual lower under various longitudinal slopes varied markedly when driving on uphill and downhill sections. The average fixation time and visual lower under the two factors were markedly different. By developing a multiple linear region model among HR, illumination, and vehicle speed at the entrance segment, Wu et al. [39] investigated the impact of external factors on HR. Regarding long and steep downhill sections, Yang et al. [40] revealed that drivers’ pupils were larger on the black spot section of the long downhill road than on other road sections. Using three models, Gu [41] found that when the downhill longitudinal slope was 3.7%, the eye movement speed was the smallest, and when the downhill longitudinal slope was 3.6%, the range of the gaze point was the smallest. Chen et al. [42] pointed out that when the slope value is greater than 3%, the vertical curve section will not only affect the sight distance of drivers but also have an important impact on the accident rate. In addition, many scholars are committed to exploring the impact of road slope, horizontal and vertical line type, and slope length on drivers’ physiological and behavioral characteristics [43,44], analyzing the change relationship between slope, horizontal and vertical line type and slope length, and physiological indicators (mainly driver HR), and finally determined the relevant indicators of drivers’ comfortable expressway alignment. For instance, when studying the impact of slope on driving load, Xu et al. [44] found that the driving speed increases with tunnel slope when driving downhill but decreases with an increase in speed when driving uphill. However, the HR growth of drivers increases with longitudinal slope, whether uphill or downhill.

### 2.3. Slope Length and Drivers’ Physiological and Behavioral Characteristics

In road alignment design, slope length design also needs to be considered. When driving on a longitudinal slope, if the slope is too long, the driver needs to accelerate or brake to control the speed in time to ensure the smooth passage of the vehicle. When the slope is too short, due to terrain constraints, there will be many slope change points on the road. When driving on a slope for a long period, drivers experience a sense of weight loss or overweight, psychological discomfort, and even bumps and dizziness, affecting driving safety. In particular, because of the effect of slope length on driving performance and psychological discomfort, mountain roads are accident-prone areas [45]. When analyzing the relationships between mountain road slope length, speed, and driver HR, Wang [46] found that there was no significant correlation between slope length and driver HR, but there was a significant correlation with speed. There were differences in the results obtained by Qiao et al. [43] through a real vehicle experiment on mountain roads, with the driver’s HR significantly correlated with slope length and slope and the driver’s respiratory rate was directly proportional to the slope length. Lin (2013) pointed out that when driving downhill, there is an obvious positive correlation between the driver’s driving load change and the longitudinal slope length. In addition, Wei et al. [9] also showed that there was a significant correlation between slope length and driver HR. In addition, they also proposed corresponding slope length limit values for various slopes.

### 2.4. Tunnel Height and Safety

For the influence of tunnel height on tunnel safety, the collision mechanism between ultrahigh vehicles and bridges or tunnels is studied via monitoring devices and the influence of tunnel height on critical wind speed [47]. For instance, Xiong et al. [48] proposed a vehicle superelevation monitoring and early warning system, which is installed at the upper end of tunnels and can monitor the superelevation of vehicles passing through tunnels. Gao et al. [49] introduced the concept of the tunnel aspect ratio, which defines the characteristic diameter of a fire source and provides a new formula for calculating the critical wind speed of tunnel fires.

The height limit of urban underpass tunnels in China is based on the relevant standards of highway tunnels. When the height limit is high, to avoid excessive road slopes, a long uphill and downhill length is needed, which requires substantial urban land construction. When the height limit is low, the trafficability will be limited, which will affect the comfort of drivers.

The above research mainly focused on drivers’ physiological and behavioral characteristics and tunnel safety, as well as the impact of linear design on drivers’ behavioral and physiological characteristics during driving, which makes an important contribution to road safety. However, thus far, the road parameter design schemes of urban underpass tunnels are based on the relevant standards of highway tunnels and urban roads (including the longitudinal slope, slope length, and tunnel height), and there is a lack of design specifications for urban underpass tunnels. For the road design parameters of urban underpass tunnels, there is little exploration of the “rationality” of the existing road from the perspective of driver comfort. Based on this, this paper focused on the difference in driver comfort while driving through urban underpass tunnels under various linear designs, which will help effectively analyze the impact of various factors on tunnel driving safety and improve the design of urban underpass tunnels. It plays an important role in relieving the driving pressure in tunnels and improving driver safety in urban underpass tunnels, and it can reduce the traffic accidents caused by excessive load and misoperation due to unreasonable tunnel design. From a theoretical point of view, its significance is a supplement to the theoretical research on highway tunnel traffic safety and can improve the traditional tunnel design code. From a practical point of view, it can provide support to urban traffic, highways, construction, municipal managers, and traffic participants and reduce the occurrence of traffic accidents, secondary accidents, and environmental pollution.

Specifically, the purpose of this paper is to explore the structural parameter optimization of urban underpass tunnels based on drivers’ physiological and behavioral comfort. Physiological indexes, such as driver HR, HR variability, and HR growth rate, are generally used in research on drivers’ physiological load [50,51,52], indicating that the HR index can reflect the impact of vehicle running speed, highway geometry, and road environmental characteristics on the driver’s physiological index while driving well. Moreover, HR data are easy to collect. Therefore, based on the driving simulator experiment, taking the driver’s HR and RR interval as physiological indicators and speed and lane centerline offset as behavioral indicators, this paper investigates the physiological and behavioral characteristics of drivers in urban underpass tunnels, focusing on the following:

1. Exploring the effects of various tunnel structural parameters on drivers’ physiological and behavioral characteristics.
2. Quantifying the relationship between slope, slope length, and tunnel height and driver physiology (HR, RR gap) and behavior (speed, lane centerline offset)
3. The optimal combination of structural parameters for urban underpass tunnels was determined by using the comfort thresholds of drivers’ physiological and behavioral indexes.

The goal is to realize design optimization of the structural parameters for urban underpass tunnels from the perspective of driver physiological comfort, improve the existing urban underpass tunnels, and provide a certain theoretical basis for the design of urban underpass tunnels in the future.

## 3. Methodology

### 3.1. Simulation Scenario

#### 3.1.1. Apparatus

**Figure 1:**
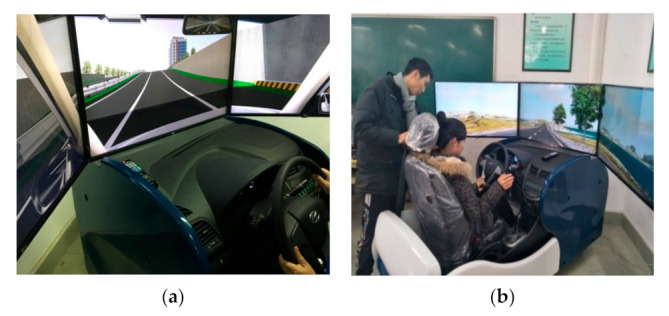
Driving simulator. (**a**) Driving simulator. (**b**) Driving simulator pre-test scenario.

The study was conducted using a SCANeR^TM^ studio [53] version 1.6 driving simulator. The hardware is composed of four networked computers, one that processes motion equations and three that generate images (Figure 1). The data recording system measured all objective parameters (including the relative position with respect to the road axis, local speed, acceleration, steering wheel rotation angle, and pedal movements).

The driving simulator was positioned in front of three angled screens in a semicircular array. This setup provided a realistic view of the road and the surrounding environment [54]. The entire projection image produced a 150° horizontal ×50° vertical forward view of the simulated roadway from the driver’s seat. The resolution of each visual scene was 1920×1080 pixels. The driver’s cab included a seat, accelerator and brake pedals, steering wheel, joystick, instrument display disk, gear shift, hand brake, and rearview mirror; these features were designed to match those of a real car. The brake, accelerator, and clutch pedals of the vehicle operate in a realistic manner. Similarly, the instrument panel and rearview mirror display vehicle speed information and peripheral vision traffic conditions in real time.

**Figure 2:**
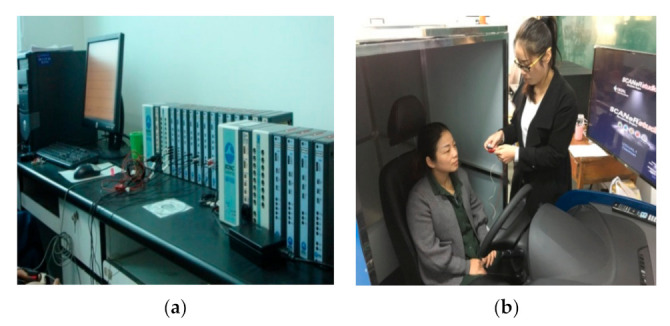
Physiological recorder (**a**) Physiological recorder. (**b**) Scene of wearing physiological recorder.

In the experiment, an MP150 model 16-channel multichannel physiological recorder [55] (see Figure 2) was used to determine electrocardiogram changes when passing through the simulated urban underpass tunnel. During simulation, the signal wires of the connecting electrode plates were attached to the left and right chest and abdomen of the driver. The software Acqknowledge was used to record and analyze the data.

#### 3.1.2. Selection of the Structural Parameters of the Simulated Urban Underpass Tunnels

Based on the CJJ211-2015 design standard of urban underground road engineering [56] and the values used in practice, three parameter thresholds for tunnels built under cities were selected. The change interval in the tunnel slope was set to 3–7%. The change interval in the uphill and downhill slope lengths was set to 80–120 m. The change interval in the tunnel height was set to 3.6–4.4 m. Further details are given in Table 1. 

In this paper, a total of 125 urban underpass tunnels were designed based on the combinations of “slope × slope length × tunnel height”. To eliminate distractions, passing, and other factors that might interfere with the drivers, the experimental road did not include other vehicles or pedestrians. In addition, through several pre-experiments, it found that most of the drivers became fatigued after driving for approximately half an hour because of the relatively empty roads. Therefore, the 125 urban underpass tunnels were divided into 5 groups. The driving time on each road was controlled to be under 25 min.

**Figure 3:**
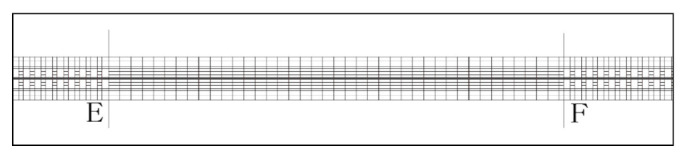
Simulated tunnel section. Note: E indicates the tunnel entrance and F indicates the tunnel exit.

The Chinese highway tunnel design specification JTG D70-2004 [57] notes that it is necessary to avoid the coupling effect of horizontal and longitudinal curves in tunnels. Most tunnels built on actual roads strictly follow this rule. To eliminate the influence of horizontal curves on driving, the horizontal curvature of the simulated urban underpass tunnels was set to 0 (i.e., a straight line). In addition, the road lines in the tunnel were solid white, in accordance with the relevant specifications (Figure 3).

**Figure 4:**
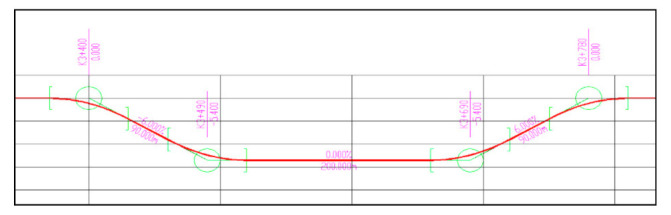
A longitudinal section of a tunnel. Note: The slope length of the tunnel was set to 90 m, the slope was set to 6%, and the length of the inner section of the tunnel was set to 200 m.

The length of each urban underpass tunnel was set to approximately 400 m, according to the structural parameters shown in Table 1. The length of the inner section of the tunnel was set to 200 m. The uphill and downhill sections of each urban underpass tunnel had exactly the same parameters. A longitudinal section of a tunnel is illustrated in Figure 4.

**Figure 5:**
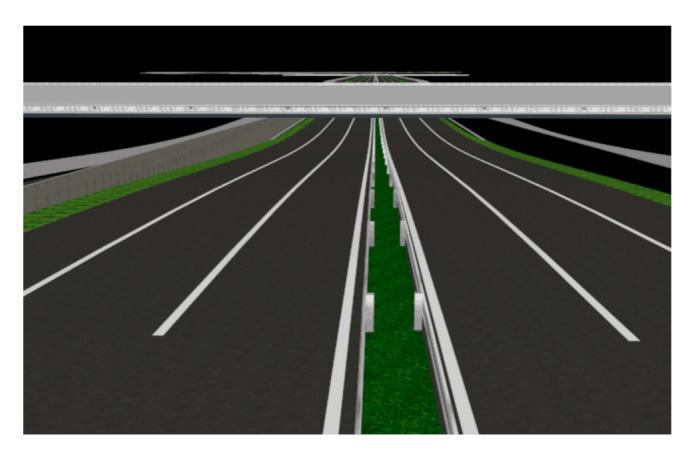
Example of a 3DMAX urban underpass tunnel.

After adding the major lines to the urban roads, 3DMAX software was used to map and render the roads following normal specifications and requirements (such as marking lines, green belts, and median barriers). The tunnels were rendered stereoscopically to make the experimental scene as realistic as possible, improving the authenticity and generalizability of the experiment (Figure 5).

#### 3.1.3. Experimental Road

In this experiment, Weft software was used to randomly distribute 125 urban underpass tunnels across 5 urban roads, each of which was approximately 31 km long and included 25 tunnels. The road cross-section was designed to be a bidirectional 6-lane road with a width of 3.75 m and a central divider. An 800 m transitional section was included between the exit of one tunnel and the entrance to the next tunnel.

### 3.2. Participants

Thirty participants with a Chinese driver’s license were recruited. The participants were required to have had their license for at least two years and needed to have normal or corrected-to-normal vision. Additionally, the drivers were required to have a rich driving experience, with a total mileage driven of over 50,000 km, including city driving and experience with passing through an urban underpass tunnel. In total, 22 male drivers and 8 female drivers were selected. The drivers ranged in age from 31 to 60 years old (with an average of 43.97 and a standard deviation of 9.43). The driving years ranged from 5–35 years (with an average of 16.1 and a standard deviation of 10.13). Each participant was paid 200 yuan for their participation. Upon their arrival in the laboratory, each participant was briefed on the requirements of the experiment, and all read and signed an informed consent document.

### 3.3. Procedure

**Figure 6:**
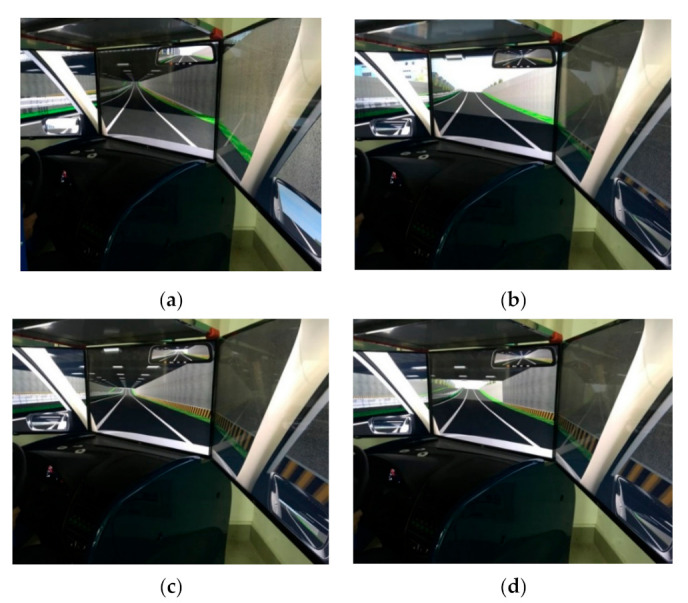
Tunnel simulation pictures. (**a**) Tunnel entrance, (**b**) Tunnel exit, and (**c**,**d**) Interior of the tunnel.

Before beginning the experiment, the experimental equipment had to be installed and debugged. After confirming that the equipment was running normally, each subject was allowed to perform adaptive driving for approximately 10 min. Once the subject was familiar with the simulator, he or she was also assumed to understand the experimental process. After the subject had demonstrated that they understood the experimental requirements, the physiology instrument patch was affixed, and observation of physiological changes began. After the data had stabilized, the data recording system of the driving simulator began logging. The subjects were required to maintain the speed limit of 80 km/h while driving freely along the middle lane (Figure 6).

When a driver passed through the entrance and exit of an urban underpass tunnel, the physiological tester analysis software recorded the signal for later data filtration. The duration of each group of experiments ranged from 22 to 25 min. At the end of each group of experiments, the simulator and physiological instrument data were saved. The drivers were then given a break of approximately 10 min to avoid fatigue. They then proceeded on the second simulated road and continued to the next set of experiments in a sequence of five roads in total.

After completing the experiment, the participants completed a demographic information questionnaire and a driving simulator dizziness status scale.

### 3.4. Data Collection

In this experiment, the MP150 model 16-channel multichannel physiological recorder was used to collect data from the time at which the driver entered the tunnel (the beginning of the downhill portion) to the time they exited the tunnel (the end of the uphill portion). The data included the HR and RR interval. The frequency was 50 Hz. The MP150 model 16-channel multichannel physiological recorder is a computer-based data acquisition system, which can collect and analyze various electrophysiological signals, such as ECG, EEG, and EMG. It is widely used in physiological signal measurement [58,59].

The driving simulator recorded real-time driving data, including time, coordinates, speed, acceleration, accelerator pedal force, brake pedal force, and lane centerline offset. The frequency of the collected data was 100 Hz. Because the tunnel entrance and exit were downhill and uphill, respectively, the driver’s change in speed at these segments was significant. Therefore, vehicle speed and lane centerline offset data were extracted separately for the uphill and downhill sections of the urban underpass tunnels.

## 4. Results

### 4.1. Partial Correlation Analysis

By plotting a scatter plot, it was demonstrated that a driver’s HR, RR interval, speed, and lane centerline offsets are related to the structural parameters of the urban underpass tunnels. To further describe this relationship, a Pearson partial correlation analysis between the driver’s HR, RR interval, speed, and lane centerline offsets and the tunnel height, slope, and slope length was conducted (Table 2).

Table 2 shows that the slope length and tunnel height are significantly negatively correlated with the driver’s HR; the slope is significantly positively correlated with the driver’s HR. The slope length and slope are significantly negatively correlated with the RR interval and the uphill speed, and the tunnel height is significantly positively correlated with the RR interval and the uphill speed. The slope length and tunnel height are also significantly positively correlated with the downhill speed, and the slope is significantly negatively correlated with the downhill speed. The slope, slope length, and tunnel height are significantly positively correlated with the uphill lane centerline offsets. Both the slope and tunnel height are significantly positively correlated with the downhill lane centerline offsets. The slope length is significantly negatively correlated with the downhill lane centerline offsets.

In general, the partial correlation analysis results demonstrate that a driver’s HR, RR interval, speed, and lane centerline offsets are significantly related to tunnel height, slope, and slope length.

### 4.2. Spatial Structure Model for Urban Underpass Tunnels

The previous section (Section 4.1) showed that drivers’ HR, RR interval, speed, and lane centerline offsets are affected by tunnel height, slope, and slope length. To further quantify the relationship between these physiological and structural variables, we used SPSS software to fit regressions between the structural parameters (tunnel height, slope, and slope length) and driver HR, RR interval, speed, and lane centerline offsets. The tunnel height, slope, and slope length were used as the independent variables (where the tunnel height is *h*, in m; slope length is *l,* in m; and the slope is *i*, in %). The driver’s physiological characteristics and vehicle operation information were used as the dependent variables (where the driver’s HR is *R*, in times/minute; the RR interval is *RR,* in s; the speed is *V,* in km/h; and the lane centerline offsets are *S*, in m).

#### 4.2.1. Model of Drivers’ HR and Structural Parameters of Urban Underpass Tunnels

Table 3 shows the results of a stepwise regression between HR and tunnel height, slope length, and slope.

According to the adjusted $R^{2}$ of 0.686, the model is significant overall; the regression formula is as follows:

(1) R=100.02−3.262h+107.4i

The regression coefficients shown in Equation (1) indicate that driver HR is negatively correlated with tunnel height and slope length but positively correlated with slope. In other words, driver HR decreases as the tunnel height increases and decreases as the slope length increases. Thus, when the tunnel height or the slope length increases, so does driving comfort. However, when the slope increases, the driver’s HR increases, indicating greater driving pressure.

#### 4.2.2. Model of the RR Interval and Structural Parameters of Urban Underpass Tunnels

Table 4 shows the results of a stepwise regression between RR interval and tunnel height, slope length, and slope.

In the model, the adjusted $R^{2}$ is 0.741, with a *p*-value of less than 0.05. Thus, the model is significant, and the regression formula is as follows:

(2) RR=0.804+0.067h−0.001l−4.4i 

The regression coefficients of Equation (2) demonstrate that the RR interval is negatively correlated with slope and slope length but positively correlated with tunnel height. In other words, the RR interval decreases as the slope and slope length increase. Thus, when the slope length or the slope increases, so does the driving pressure. However, when the tunnel height increases, so does the RR interval, indicating greater driving comfort.

#### 4.2.3. Model of the Speed and Structural Parameters of Urban Underpass Tunnels

Table 5 shows the results of a stepwise regression for speed on the uphill and downhill sections and tunnel height, slope length, and slope.

For the model including speed on the downhill section, the adjusted $R^{2}$ is 0.425, and the model is significant. The regression formula is as follows:

(3) $$V_{d}=69.400+2.666h-40.2i$$

The regression coefficients in Equation (3) show that speed on the downhill section is positively correlated with tunnel height and slope length but negatively correlated with slope. Specifically, when the tunnel height and slope length increase, so does the speed on the downhill section. However, as the slope increases, the speed on the downhill section decreases.

For the model including speed on the uphill section, the adjusted $R^{2}$ is 0.709, with a *p*-value less than 0.05. Thus, the model is significant, and the regression formula is as follows:

(4) $$V_{u}=79.344+1.525h-0.030l-155.0i$$

The regression coefficients in Equation (4) indicate that the speed on the uphill section is positively correlated with tunnel height but negatively correlated with slope and slope length. Thus, when the tunnel height increases, so does the speed on the uphill section. However, as the slope and the slope length increase, the speed on the uphill section decreases.

#### 4.2.4. Model of Lane Centerline Offsets and Structural Parameters of Urban Underpass Tunnels

Table 6 shows the results of a multiple linear regression on the lane centerline offsets of the uphill and downhill sections and tunnel height, slope length, and slope.

For the model including the lane centerline offsets on the downhill section, the adjusted $R^{2}$ is 0.399, with a *p*-value less than 0.05. Thus, the model is significant, and the regression formula is as follows:

(5) $$S_{d}=0.666-0.031h-0.001l+3.6i$$

Equation (5) shows that the lane centerline offsets on the downhill section are positively correlated with slope but negatively correlated with tunnel height and slope length. In other words, when the tunnel height and slope length increase, so does the driver’s control, but when the slope increases, the driver’s control of the vehicle decreases.

For the model including the lane centerline offsets on the uphill section, the adjusted $R^{2}$ is 0.694. Thus, the model is significant, and the regression formula is as follows:

(6) $$S_{u}=0.447-0.025h+0.001l+3.3i$$

Equation (6) for the model including the lane centerline offsets on the uphill section shows that the lane centerline offsets of the uphill section are positively correlated with slope length and slope but negatively correlated with tunnel height. In other words, when the tunnel height increases, so does the driver’s control, but when the slope and slope length increase, the driver’s control decreases.

### 4.3. Optimization of the Structural Parameters of Urban Underpass Tunnels

From Section 4.2, the quantitative relationship between the driver’s HR, RR interval, uphill and downhill speed, uphill and downhill lane centerline offsets, and the tunnel height, slope, and slope length can be obtained, as follows:

$$\begin{array}{l} R=100.02-3.262h+107.4i\\ RR=0.804+0.067h-0.001l-4.4i\\ V_{d}=69.400+2.666h-40.2i\;\\ V_{u}=79.344+1.525h-0.030l-155.0i\\ S_{d}=0.666-0.031h-0.001l+3.6i\\ S_{u}=0.447-0.025h+0.001l+3.3i \end{array}$$

A good road structure parameter should allow the driver to drive without pressure and can enable normal driving performance. Due to the special spatial structure of urban underpass tunnels, drivers experience a relatively narrow lane environment, gradually changing tunnel wall height, gradually narrowing driving space, and limited sight distance. These external environmental factors aggravate the impact of tunnel structural parameters on drivers to a certain extent, increase drivers’ tension, and imbalance the distribution of psychological resources. When driving in the downhill section of the tunnel entrance, the speed increases rapidly due to the acceleration generated by gravity. In addition, the gradually changing driving environment will make the driver have a nervous psychology, and often take braking measures to reduce the speed, so as to alleviate his inner uneasiness. When passing through the tunnel entrance and exit, the illumination difference is too large, which will directly have a great impact on the driver’s vision. When the driver is driving inside the tunnel, because the tunnel is a semi-closed environment, the driver’s psychology will obviously produce a sense of depression, which will lead the driver to want to leave the tunnel quickly, which may increase the driving speed. When driving in the uphill section of the tunnel exit, due to the limited sight distance, the gravity produces deceleration, and the driver accelerates on the slope, resulting in an increase in speed. Drivers experience different behavior, physiological, and psychological changes in the whole tunnel driving process. If this physiological and psychological change exceeds the range that the driver can bear, it is very easy to cause traffic accidents. Therefore, comfortable physiological and behavioral conditions are necessary to ensure the normal driving of drivers. Medical research has indicated that heart rate is an important index for evaluating the state of cardiovascular function, and bradycardia or tachycardia are adverse to driving. Healthy people should have a heart rate of 60~100 beats/min in a calm state [60,61,62]. The greater the value, the greater the psychological pressure and the lower the physiological comfort during driving. For the RR interval, medicine revealed that the value of the normal RR interval is 0.6~11 s. The smaller the value, the more nervous the driver is, resulting in a reduction of driving physiological comfort. Thus, the driver’s physiological constraint condition is 60≤R≤100, 0.6≤RR≤1.

Regarding speed, too high or too low speed will have an adverse impact on normal driving to a certain extent. In order to ensure the driver’s comfort when driving through the urban underpass tunnel, 15% and 85% of the vehicle speed was selected as the speed comfort threshold [63], and it can be obtained that the comfortable speeds for the drivers ranged from 77–79 km/h on the downhill section and 72–78 km/h on the uphill section. Thus, the speed constraint condition on the downhill section and uphill section is $77\leq V_{d}\leq 79$ and $72\leq V_{u}\leq 78$, respectively.

A driver in a normal state will always operate the steering wheel in time according to the degree of lane centerline offsets to ensure that the vehicle will not deviate from the lane. For the lane centerline offset, if the driver deviates too much from the lane centerline, normal driving will be affected. Therefore, to ensure that a vehicle stayed within the lane, 0–0.6 m was selected as the appropriate range for both the uphill and downhill slope sections. Thus, the lane centerline offset constraint condition on the downhill section and uphill section is $0\leq S_{d}\leq 0.6$ and $0\leq S_{u}\leq 0.6$, respectively.

Based on Equations (1)–(6) from the previous section, combined with the physiological and behavioral comfort thresholds of the drivers, we derived the following constraint models:

$$\begin{array}{cc} R=100.02-3.262h+107.4i & \;60\leq R\leq 100\\ RR=0.804+0.067h-0.001l-4.4i & \;0.6\leq RR\leq 1\\ \;V_{d}=69.400+2.666h-40.2i & \;77\leq V_{d}\leq 79\\ V_{u}=79.344+1.525h-0.030l-155.0i\; & 72\leq V_{u}\leq 78\\ S_{d}=0.666-0.031h-0.001l+3.6i\; & 0\leq S_{d}\leq 0.6\\ S_{u}=0.447-0.025h+0.001l+3.3i\; & 0\leq S_{u}\leq 0.6 \end{array}$$

These constraint models showed the following ranges for the variables: 0.2% for slope, 0.2 m for tunnel height, and 10 m for slope length. Next, parameter thresholds were obtained based on driver physiological and behavioral comfort levels. Specifically, the optimal slope ranged from 1.4 to 5.4%, the slope length ranged from 60 to 180 m, and the tunnel height ranged from 3.6 to 4.4 m. Finally, the optimal structural parameter combinations for the 237 groups of urban underpass tunnels were determined, as shown in Table 7.

## 5. Discussion

The purpose of this study was to explore the effects of various structural parameters of urban underpass tunnels on drivers’ physiological and behavioral comfort. By exploring the physiological and behavioral change characteristics of drivers navigating urban underpass tunnels, the relationships between various combinations of structural parameters and the drivers’ physiological and behavioral indicators were quantified, and based on the drivers’ physiological and behavioral comfort thresholds, the structural parameter combinations of urban tunnels that can make drivers feel comfortable were deduced. This paper makes two main contributions. The first involves considering the influence of urban underpass tunnel design from the perspective of human factors, more specifically from the perspective of the driver’s physiological comfort, and identifying the relationship between structural parameters and drivers’ physiological and behavioral parameters. The other contribution is determining the combination of structural parameters based on the driver’s physiological and behavioral comfort threshold by quantifying the relationship between drivers’ physiological and behavioral indicators and urban underpass tunnels and further by determining drivers’ physiological and behavioral comfort thresholds. From the perspective of driver comfort, this study realizes the design optimization of structural parameters of urban underpass tunnels, improves the existing urban underpass tunnel, and provides a certain theoretical basis for the design of urban underpass tunnels in the future.

With regard to research on urban underpass tunnels, some scholars have explored the impact of urban tunnels on driver vision, physiology, and speed by carrying out real vehicle experiments [64,65]. However, this paper obtained data via a driving simulation experiment, as it mainly explored the impact of various structural parameters of urban underpass tunnels on driver physiology and behavior. Using a driving simulator, various combinations of structural parameters that are difficult to obtain on real roads were acquired to explore the impact of road design on drivers’ physiological comfort. In addition, the behavioral effectiveness of driving simulators has also been verified by many researchers [30,66]. The advantage of being able to manipulate scenarios and confounding variables enabled a repetitive reproduction of conditions across participants without putting them in danger, which was an invaluable asset for addressing a wide range of questions. For the sake of controllability, repeatability, and safety, driving simulators are extensively used for driver behavior research.

First, the research methods for driving behavior and physiology have been discussed in the article. Specifically, the heart and brain are constantly communicating. For instance, when we encounter dangerous situations, signals from the brain can make the heart beat faster. When you relax, your heart slows down. Researchers from institutions, such as the Institute of Human Cognition and Brain Science of the German Max Planck Society, have confirmed two mechanisms by which the heart affects perception and claim that the perception of external stimuli changes with heartbeat [67]. Vanderhaegen et al. [68] discussed the synchronization between dynamic events with heartbeats and its impact on non-conscious errors in the control of dynamic events. Due to the special spatial structure of urban underpass tunnels, drivers experience a relatively narrow lane environment, gradually changing tunnel wall height, gradually narrowing driving space, and limited sight distance. These external environmental factors aggravate the impact of tunnel structural parameters on drivers to a certain extent, increase drivers’ tension, and imbalance the distribution of psychological resources. As a result, the psychological load increases, and the driver’s physiological comfort decreases [69,70]. According to the inverted U-shaped curve relationship between human workload and performance, an appropriate load state plays an important role in safe driving [71]. However, excessive load slows drivers’ responses, increases the probability of decision-making errors, and significantly increases the risk of traffic accidents [72]. Accident statistics show that driver factors account for approximately 93% of the total number of accidents [73]. Therefore, it is necessary to study the spatial variation characteristics of drivers’ psychological loads in urban tunnel environments from the perspective of drivers and explore the internal mechanism driving behavior change. Drivers’ psychological load measurement methods mainly include subjective evaluation methods, main task performance methods, dual task performance methods, and physiological parameter evaluation methods [74,75]. The subjective evaluation method mostly adopts the form of an evaluation scale or questionnaire. Commonly used scales, such as NASA-TLX, WP, and SWAT, require subjects to self-evaluate the load [76]. The performance measurement method analyzes the driving performance of subjects in the process of completing a single task or dual task, such as response time to a stress scene, lane change performance, driving trajectory deviation, and other indicators [77,78], judging the degree of overall performance decline during task execution. In addition, the emotional state of drivers could be assessed by using subjective methods, such as Self-Assessment-Manikin [79] or the Reverse Comic Strip [80]. Physiological parameters are measured based on changes in EEG, ECG, eye movement, and other indicators of drivers [81,82,83,84], indirectly evaluating their psychological loads. Among them, the subjective evaluation method is greatly affected by individual differences and cannot evaluate driving performance and load in real time. In comparison, in a simulated driving situation, the physiological parameter evaluation method is used to collect a driver’s physiological indicators and behavior indicators and, on this basis, to evaluate the driver’s physiological comfort and work performance, which is closer to and reflects the driver’s real state.

The changes in the HR, RR interval, speed, and lane centerline offsets of drivers in tunnels with various combinations of structural parameters (slope, slope length, and tunnel height) have also been mentioned by other scholars. For instance, Zhao et al. [85] studied the effect of the longitudinal slope of highway tunnels on a driver’s HR. They found that when driving on the uphill sections, vehicle speed increased with slope, and while driving on the downhill sections, the speed first increased and then decreased with increasing slope. Similarly, when driving on the downhill sections, the driver’s HR first increased and then decreased as the slope increased. A similar study also examined stretches of highways with a long slope. Yan [86] found that when drivers were driving on the uphill and downhill sections, the driver’s HR and vehicle speed both increased at first and then decreased with increasing slope. In addition, in the study of a mountainous highway with a long slope, the authors found that greater slopes led to greater changes in the HRs of drivers, resulting in a parabolic relationship between the slope length and driver HR. Drivers’ HRs first decreased and then increased with slope length. Thus, the relationships between a driver’s HR, driving speed, slope length, and slope vary by driving environment. Specific environmental characteristics (such as the carriage environment, illumination, tunnel walls, and driving space) may alter the relationship between driver HR, speed, slope, and slope length for highway tunnels, long sloped sections of highway, and uphill and downhill sections of a mountain highway. This study provides a new theoretical basis for the design of urban underpass tunnels with various structural parameters.

Finally, more importantly, this paper not only analyzed the effects of various structural parameters on drivers’ physiology and behavior but also determined the drivers’ physiology and comfort thresholds. According to the quantitative relationship between structural parameters and drivers’ physiological and behavioral indexes, the optimal combination of structural parameters based on drivers’ physiological and behavioral comfort was deduced by using the constraint relationship of physiological and comfort thresholds. Combined with ergonomics, this paper analyzed the optimal parameters of urban underpass tunnels from the driver’s point of view, which can reasonably improve the tunnel design in China, ensure tunnel traffic safety, and seek the maximum comfort of the driver in the driving process.

Some limitations should be addressed by future research. In this experimental design, the height, slope, and slope length of urban underpass tunnels were taken as independent variables, and other variables related to the tunnel were controlled for. In a follow-up study, other influencing factors, such as light intensity, road width, and traffic density, should be integrated. The driving simulation test was conducted under good weather conditions. Therefore, in subsequent field research, the impact of bad weather (such as fog, snow, and rain) or extreme conditions on driver performance should be considered. In addition to the physiological and behavioral characteristics of drivers, it is also necessary to thoroughly analyze the impact of tunnel structural parameters on other drivers’ behaviors (such as the driver’s vision). In addition, the current research was based on small sample experimental tests. Large-scale experiments should be carried out in a real environment to further clarify the impact of tunnel structural parameters on driver driving comfort.

## 6. Conclusions

Based on experiments in a driving simulator, this paper focused on the relationship between driver HR, RR interval, speed, and lane centerline offsets and the tunnel height, slope, and slope length of urban underpass tunnels. The effects of these structural parameters on the physiological and behavioral characteristics of drivers were analyzed. The following conclusions were drawn:

Driver HR, RR interval, speed, and lane centerline offsets were significantly related to tunnel height, slope, and slope length.

The quantitative relationship between structural parameters and drivers’ physiological and behavioral indexes and the optimal combination of structural parameters based on drivers’ physiological and behavioral comfort were deduced by using the constraint relationship of physiological and comfort thresholds.

This study is significant from a theoretical perspective because it builds on studies of highway tunnel traffic safety and has implications for improving traditional tunnel design specifications. From a practical point of view, the results can be useful for urban transportation, highways, and construction, helping municipal managers and drivers reduce traffic accidents (including secondary accidents) and environmental pollution. In general, optimizing the design of urban underpass tunnels based on the physiological and behavioral comfort of drivers can increase road safety.

## Figures and Tables

**Table 1:** Structural parameters of urban underpass tunnels.

Slope (%)	Slope Length (m)	Tunnel Height (m)
3	80	3.6
4	90	3.8
5	100	4
6	110	4.2
7	120	4.4

**Table 2:** Partial correlation analysis between driver HR, RR interval, speed, and lane centerline offsets and urban underpass tunnels with different structural parameters.

Control Variables	Variables	HR	RR Interval	Uphill Speed	Downhill Speed	Uphill Lane Centerline Offsets	Downhill Lane Centerline Offsets
h×i	*l*	−0.095 *	−0.35 ***	−0.299 ***	0.135 *	0.276 **	−0.018 *
h×l	*i*	0.788 ***	−0.848 ***	−0.841 ***	−0.467 ***	0.83 ***	0.623 ***
i×l	*h*	−0.623 ***	0.442 ***	0.296 ***	0.595 ***	0.219 *	0.183 *

* *p* < 0.05, ** *p* < 0.01, and *** *p* < 0.001. h represents tunnel height; i represents slope; l represents slope length.

**Table 3:** Parameters for regression of heart rate and structural parameters.

Model	Unstandardized Coefficients		*t*	Sig.
	*B*	Standard Error		
Constant	100.02	1.574	63.555	0.000
Tunnel height (h)	−3.262	0.381	−8.566	0.000
Slope (i)	107.4	0.076	14.107	0.000

Sig. means significance; *p*-level (Sig.) < 0.05, indicating statistical significance.

**Table 4:** Parameters for regression of the RR interval and structural parameters.

Model	Unstandardized Coefficients		*t*	Sig.
	*B*	Standard Error		
Constant	0.804	0.057	14.123	0.000
Tunnel height (h)	0.067	0.012	5.421	0.000
Slope length (l)	−0.001	0.000	−4.114	0.000
Slope (i)	−4.4	0.002	−17.624	0.000

**Table 5:** Parameters for regression of speed on the uphill and downhill sections and structural parameters.

Model	Unstandardized Coefficients		*t*	Sig.
	*B*	Standard Error		
Downhill model				
Constant	69.400	1.426	48.651	0.000
Tunnel height (h)	2.666	0.345	7.724	0.000
Slope (i)	−40.2	0.060	−5.824	0.000
Uphill model				
Constant	79.344	2.083	38.089	0
Tunnel height (h)	1.525	0.454	3.416	0.001
Slope length (l)	−0.030	0.009	−3.263	0.001
Slope (i)	−155.0	0.091	−16.800	0

*p*-Level (Sig.) < 0.05, indicating statistical significance.

**Table 6:** Parameters for regression of the lane centerline offsets on the uphill and downhill sections and structural parameters.

Model	Unstandardized Coefficients		t	Sig.
	B	Standard Error		
Downhill model				
Constant	0.666	0.081	8.265	0
Tunnel height (h)	−0.031	0.018	−2.052	0.042
Slope length (l)	−0.001	0	−2.028	0.045
Slope (i)	3.6	0.004	8.769	0
Uphill model				
Constant	0.447	0.046	9.737	0
Tunnel height (h)	−0.025	0.01	−2.469	0.015
Slope length (l)	0.001	0	3.156	0.002
Slope (i)	3.3	0.002	16.375	0

*p*-Level (Sig.) < 0.05, indicating statistical significance.

**Table 7:** Optimal structural parameters of urban underpass tunnels.

Tunnel Height (m)	Slope Length (m)	Slope (%)
3.6	70	3.2
	80	3–3.4
	90	2.8–3.6
	100	2.6–4
	110	2.4–4
	120	2.2–3.6
	130	2–3.4
	140	1.8–3
	150	1.6–2.4
	160	1.4–2.4
	170	1.2–2.2
	180	1–1.8
	190	0.8–1.6
	200	0.6–1.2
	210	0.4–1
	220	0.2–0.6
	230	0–0.2
	240	0
3.8	80	3.2–3.6
	90	3–3.8
	100	2.8–4.2
	110	2.6–4
	120	2.4–3.8
	130	2.2–3.4
	140	2–3.2
	150	1.8–2.8
	160	1.6–2.6
	170	1.4–2.2
	180	1.4–2
	190	1.4–1.6
	200	1.4
4	80	3.4–3.8
	90	3.2–4
	100	3–4.2
	110	2.8–4.2
	120	2.8–4
	130	2.8–3.6
	140	2.8–3.4
	150	2.8–3
	160	2.8
4.2	80	4
	90	4–4.2
	100	4–4.4
	110	4–4.4
	120	4

## Data Availability

The data that support the findings of this study are available from the corresponding author, upon reasonable request.
